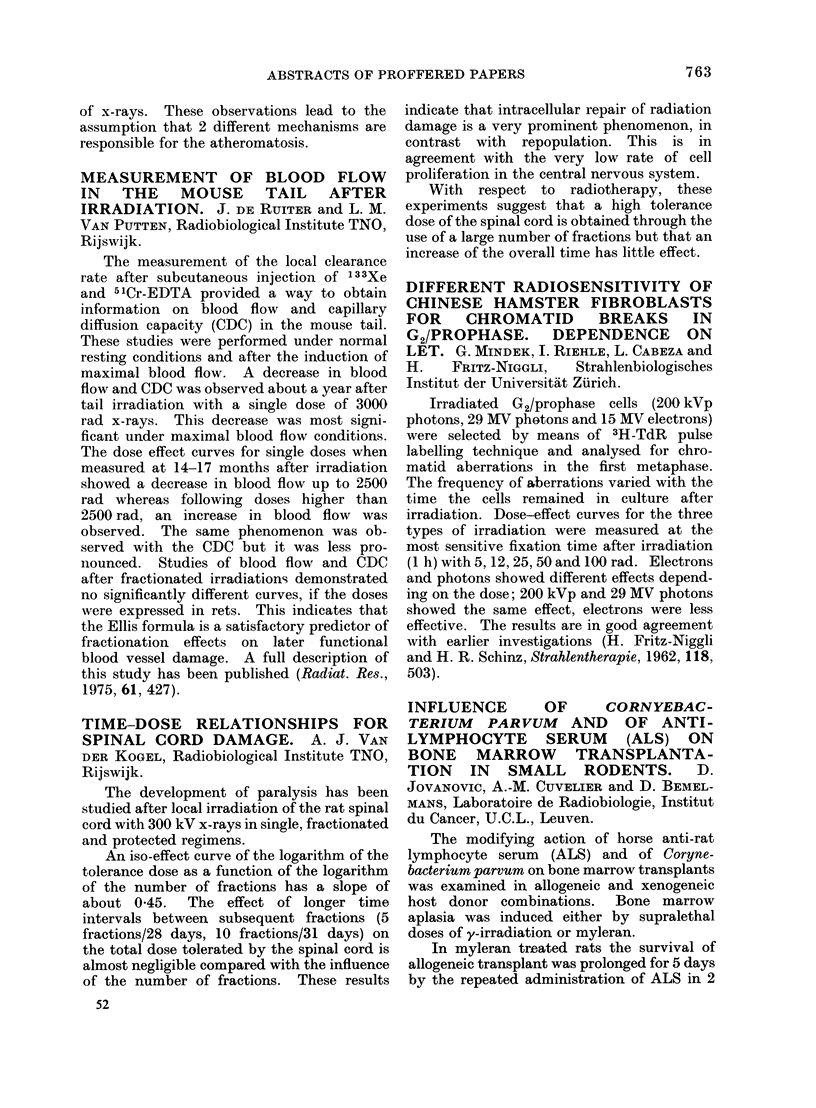# Proceedings: Measurement of blood flow in the mouse tail after irradiation.

**DOI:** 10.1038/bjc.1975.328

**Published:** 1975-12

**Authors:** J. de Rutter, L. M. Van Putten


**MEASUREMENT OF BLOOD FLOW IN THE MOUSE TAIL AFTER IRRADIATION.** J. de Ruiter and L. M. Van Putten, Radiobiological Institute TNO, Rijswijk.

The measurement of the local clearance rate after subcutaneous injection of $^{133}Xe$ and $^{51}Cr$-EDTA provided a way to obtain information on blood flow and capillary diffusion capacity (CDC) in the mouse tail. These studies were performed under normal resting conditions and after the induction of maximal blood flow. A decrease in blood flow and CDC was observed about a year after tail irradiation with a single dose of 3000 rad x-rays. This decrease was most significant under maximal blood flow conditions. The dose effect curves for single doses when measured at 14–17 months after irradiation showed a decrease in blood flow up to 2500 rad whereas following doses higher than 2500 rad, an increase in blood flow was observed. The same phenomenon was observed with the CDC but it was less pronounced. Studies of blood flow and CDC after fractionated irradiations demonstrated no significantly different curves, if the doses were expressed in rets. This indicates that the Ellis formula is a satisfactory predictor of fractionation effects on later functional blood vessel damage. A full description of this study has been published (*Radiat. Res.*, 1975, **61**, 427).